# Echocardiographic characteristics of dogs with pulmonary hypertension secondary to respiratory diseases

**DOI:** 10.1111/jvim.16836

**Published:** 2023-08-18

**Authors:** Yunosuke Yuchi, Ryohei Suzuki, Takahiro Saito, Yuyo Yasumura, Takahiro Teshima, Hirotaka Matsumoto, Hidekazu Koyama

**Affiliations:** ^1^ Laboratory of Veterinary Internal Medicine, School of Veterinary Medicine, Faculty of Veterinary Science Nippon Veterinary and Life Science University Tokyo Japan

**Keywords:** canine, myocardial strain, obstructive airway/lung disease, restrictive lung disease, right heart failure, speckle tracking echocardiography

## Abstract

**Background:**

Pulmonary hypertension (PH) secondary to respiratory disease is caused by pulmonary vascular remodeling and hypoxia. Severe PH can induce various clinical signs, including syncope and right‐sided heart failure.

**Hypothesis/Objectives:**

To investigate the echocardiographic characteristics in dogs with PH secondary to respiratory diseases.

**Animals:**

Thirty‐one dogs with respiratory diseases with or without PH and 15 healthy dogs.

**Methods:**

Prospective cross‐sectional study. Dogs were classified according to respiratory disease (obstructive airway/lung disease [OALD] or restrictive lung disease [RLD]) and PH‐relevant signs. The association between echocardiographic variables and PH (classified by respiratory disease and PH‐relevant signs) was investigated.

**Results:**

Twenty‐one dogs were diagnosed with PH; of these, 11 showed PH‐related signs (OALD, n = 2; RLD, n = 9), 14 had right ventricular hypertrophy, and 19 had pulmonary arterial enlargement. Right ventricular dysfunction and dilatation were observed only in dogs with PH‐related signs (n = 10). Left and right ventricular stroke volumes were significantly lower in dogs with PH (median [interquartile range]: 17.2 [12.4‐20.8] and 16.8 [15.3‐29.5] mL/m^2^, respectively). Dogs with RLD had higher echocardiography‐estimated pulmonary vascular resistance than those with OALD (median [interquartile range]: 3.1 [1.9‐3.3] and 1.6 [1.3‐2.2], respectively).

**Conclusions and Clinical Importance:**

Pulmonary arterial enlargement was the most common echocardiographic finding in dogs with PH secondary to respiratory diseases. Right ventricular dysfunction, dilatation, and decreased left and right ventricular stroke volume were significantly associated with the PH‐related signs, indicating that comprehensive echocardiography is recommended in dogs with respiratory disease. Restricted lung disease might induce more severe PH than OALD.

**Abbreviations**
2D‐STE: 2‐dimensional speckle tracking echocardiography
3seg: only RV‐free wall analysis
6seg: global RV analysis
ACVIM: American College of Veterinary Internal Medicine
AT/ET: acceleration time to ejection time ratio
CI: confidence interval
CVC: maximal caudal vena cava
FAC: fractional area change
LA/Ao: left atrial to aortic diameter ratio
LV: left ventricular/left ventricle
LVIDDN: end‐diastolic LV internal diameter normalized by body weight
LVIDSN: end‐systolic LV internal diameter normalized by body weight
LV‐SC: LV circumferential strain
LV‐SL: LV longitudinal strain
OALD: obstructive airway/lung disease
PA/Ao: pulmonary artery to aortic diameter ratio
PH: pulmonary hypertension
PVRecho: pulmonary vascular resistance estimated by echocardiography
RAA: right atrial area
RHF: right heart failure
RLD: restrictive lung disease
RPAD index: right pulmonary artery distensibility index
RV: right ventricular/right ventricle
RV s': peak systolic myocardial velocity at the lateral tricuspid annulus
RVEDA: end‐diastolic RV area
RVESA: end‐systolic RV area
RVIDd: end‐diastolic RV internal diameter
RV‐SL: RV longitudinal strain
RVWTd: end‐diastolic RV‐free wall thickness
SV: stroke volume
TAPSE: tricuspid annular plane systolic excursion

## 1 INTRODUCTION

Pulmonary hypertension (PH) is an intractable cardiovascular disorder in dogs characterized by increased pulmonary arterial pressure and vascular resistance.^1^ Increased pulmonary arterial pressure is associated with increased right ventricular (RV) afterload and induces RV hypertrophy, dysfunction, and dilatation. According to the American College of Veterinary Internal Medicine (ACVIM) consensus guidelines, various diseases, not just cardiovascular diseases, can induce PH.^1^ Specifically, respiratory diseases and hypoxia, or hypoxia, can cause pulmonary vascular remodeling (eg, interstitial fibrosis) and hypoxic pulmonary vasoconstriction, and finally lead to PH. In severe cases, PH might induce various clinical findings (eg, syncope and right‐sided congestive heart failure [RHF]) through excessively increased RV afterload, RV dysfunction, and decreased cardiac output.^1^ Some recent studies report clinical characteristics and worse outcomes in dogs with PH secondary to respiratory disease.^2^, ^3^, ^4^ Therefore, evaluating the pathophysiology of PH in dogs with respiratory diseases is essential for appropriate management of the condition.

Right‐heart catheterization is the gold standard for diagnosing and classifying PH in veterinary medicine. However, its invasive nature and the need for anesthesia prevent veterinary clinicians from routinely performing right‐heart catheterization in dogs and cats. Therefore, various echocardiographic variables have been used as alternatives for the diagnosis and stratification of PH in dogs, including RV area, RV internal diameters, RV fractional area change (FAC), and tricuspid annular plane systolic excursion (TAPSE).^5^, ^6^, ^7^, ^8^, ^9^, ^10^ Additionally, 2‐dimensional speckle‐tracking echocardiography (2D‐STE) allows precise RV functional assessment with a low influence on angle dependency and loading conditions.^9^, ^11^, ^12^ However, no study has evaluated precise RV function using 2D‐STE in dogs with PH secondary to respiratory disease.

Therefore, this study aimed to investigate the echocardiographic characteristics of PH secondary to respiratory disease in dogs. We hypothesized that the degree of RV morphological and functional disorders might differ depending on the classification of respiratory disease (obstructive airway/lung disease [OALD] and restrictive lung disease [RLD]).

## 2 MATERIALS AND METHODS

This study was a prospective cross‐sectional study. All procedures followed the Guidelines for Institutional Laboratory Animal Care and Use, and the study was approved by the Ethics Committee for Animal Use. Written informed consent authorized for the participation of each dog in the study was obtained from its owners.

### 2.1 Animals

Client‐owned healthy dogs and dogs with respiratory diseases in our institution who underwent echocardiography between April 2019 and December 2021 were prospectively included in our study. All dogs underwent a complete physical examination, transcutaneous oxygen saturation measurement, venous blood gas measurements, 30‐second electrocardiography (performed multiple times in all cases), blood pressure measurement by the oscillometric method, and transthoracic radiographic and echocardiographic examinations. Dogs were diagnosed as healthy based if there were no abnormalities in the above examinations. Dogs were excluded if they had diseases that could increase pulmonary arterial pressure other than respiratory diseases (eg, left heart disease, congenital disease, and thromboembolic disease), systemic diseases that could influence cardiac function (additional blood biochemistry and coagulation tests were performed as needed), systemic hypertension (systolic systemic blood pressure > 160 mmHg),^13^ and missing data, or missing data. Dogs with trivial mitral valve insufficiency that did not affect cardiac function were included in this study. The mitral valve insufficiency was determined as trivial when Doppler echocardiography showed a small jet area in the left atrium, right behind the closed valve leaflets in systole, that was not sustained in all systolic phases.^14^, ^15^

Dogs with respiratory disease were classified into 1 of the PH probability groups (low, intermediate, and high) according to the tricuspid regurgitation (TR) velocity and echocardiographic abnormalities at 3 anatomical sites described in the ACVIM consensus guideline: (1) ventricles, (2) pulmonary artery, and (3) right atrium and caudal vena cava.^1^ Dogs were considered to have no or low PH probability if they had any echocardiographic abnormalities suggestive of PH or only a TR velocity ≤ 3.0 m/s; an intermediate PH probability if they had 2 sites of echocardiographic abnormalities and a TR velocity ≤ 3.0 m/s, no or 1 site of echocardiographic abnormality and a TR velocity 3.0‐3.4 m/s, or a TR velocity > 3.4 m/s without any echocardiographic abnormalities; and a high PH probability if they had 3 sites of echocardiographic abnormalities, more than 2 sites of echocardiographic abnormalities and a TR velocity 3.0‐3.4 m/s, or more than 1 site of echocardiographic abnormality and a TR velocity > 3.4 m/s.^1^ In this study, dogs with an intermediate or high probability of PH were diagnosed with PH. Respiratory diseases were clinically classified into 2 subtypes based on previous reports: OALD and RLD.^2^, ^16^, ^17^, ^18^ The OALD is a disease that affects the extra‐ and intrathoracic trachea, bronchi, bronchioles, or some combination of these obstructing airflow; whereas RLD is a pleural or parenchymal disorder that restricts lung expansion during inspiration. The clinical diagnosis of OALD was made by thoracic radiography in the inspiratory and expiratory phases, and if necessary fluoroscopic examination. The RLD was clinically diagnosed based on radiographic and ultrasonographic findings, computed tomography findings (when possible), clinical signs, clinical course, and therapeutic response. In this study, dogs with PH were classified according to clinical signs associated with PH (PH signs), defined as the presence of RHF, syncope, or both. Dogs were clinically diagnosed as having RHF if the dog had any radiographic or ultrasonographic findings indicative of ascites, pleural effusion, and pericardial effusion, or pericardial effusion, without any abnormality other than PH that might be responsible.^8^, ^9^, ^19^ Fluid aspiration with subsequent cytologic analysis was performed whenever possible to prove the effusions were transudative.

### 2.2 Echocardiography

Two‐dimensional and Doppler echocardiography was performed by a single investigator using the Vivid E95 Ultra Edition echocardiographic system (GE Healthcare, Tokyo, Japan), with simultaneous ECG recordings. All data were recorded from nonsedated dogs manually restrained in the right and left lateral recumbencies for at least 5 consecutive cardiac cycles. All echocardiographic measurements were performed by an EchoPAC version 204 workstation (GE Healthcare, Tokyo, Japan) by another blinded observer trained by a cardiologist. The mean values obtained from 3 consecutive cardiac cycles of sinus rhythm were used for statistical analyses.

The left atrial to aortic diameter ratio (LA/Ao), end‐diastolic and end‐systolic left ventricular (LV) internal diameters normalized by body weight (LVIDDN and LVIDSN, respectively), and fractional shortening were measured for morphological and functional indicators of the left heart. For RV morphological indicators, the end‐diastolic and end‐systolic RV areas (RVEDA and RVESA, respectively), end‐diastolic RV internal diameter (RVIDd), and end‐diastolic RV‐free wall thickness (RVWTd) were obtained by the left apical 4‐chamber view optimized for the right heart (RV focus view).^20^, ^21^, ^22^ These morphological variables were indexed by body weight as previously described.^6^, ^7^, ^20^ The right atrial area was additionally measured by tracing the endomyocardial border of the right atrium at end‐systole in the RV focus view and was normalized for body weight (RAA index) as previously described.^21^ To evaluate the pulmonary artery, the pulmonary artery to aortic diameter ratio (PA/Ao) and right pulmonary artery distensibility (RPAD) index were measured from the right parasternal short‐axis view at the level of the pulmonary artery by previously described methods.^23^ The RPAD index was calculated by the right pulmonary artery diameter (RPA) in the minimal (at the Q wave) and maximal (at the largest T wave deflection) phases: RPAD index = (maximal RPA − minimal RPA)/maximal RPA × 100.^23^ Furthermore, maximum caudal vena cava diameter normalized for body weight (CVC index) was obtained by the left cranial parasternal view as previously described.^21^ As functional indicators of RV, RV FAC, B‐mode method‐derived TAPSE, and tissue Doppler imaging‐derived peak systolic myocardial velocity at the lateral tricuspid annulus (RV s') were obtained. The normalized values of RV FAC and TAPSE (RV FACn and TAPSEn, respectively) were calculated by the formulas described in previous reports.^19^, ^20^, ^24^ Additionally, LV and RV stroke volumes normalized for body surface area (SV) were obtained by multiplying the velocity‐time integral of the aortic or pulmonary artery flow and the cross‐sectional area of the aortic or pulmonary trunk, as previously described.^25^, ^26^ The cross‐sectional areas of the aortic and pulmonary trunk were obtained from the right parasternal long‐axis view optimized for the LV outflow tract and the right parasternal short‐axis view at the level of the pulmonary artery, respectively. Furthermore, pulmonary vascular resistance estimated by echocardiography (PVRecho) was calculated by dividing the square of the TR velocity (m/s) by the velocity‐time integral of pulmonary artery flow (cm), as previously described.^19^, ^27^, ^28^ The TR was obtained by inspecting the tricuspid valve from multiple views, and the TR velocity was defined as the highest velocity obtained from the dense TR flow profile. This study determined echocardiographic abnormalities regarding ventricles, pulmonary artery, right atrium, and caudal vena cava according to the qualitative and quantitative echocardiographic findings described in the ACVIM consensus for assessing PH probability.^1^ Enlargement of the RV, right atrium, and caudal vena cava were defined as exceeding the upper limit of reference intervals by allometric scaling in healthy dogs, as previously reported.^21^ Right ventricular dysfunction was determined based on any 1 of RV FAC, TAPSE, and RV s' below the described reference intervals.^20^, ^21^

### 2.3 Two‐dimensional speckle tracking echocardiography

The 2D‐STE was performed by the same workstation as that used for echocardiographic measurements.^9^, ^26^, ^29^, ^30^, ^31^ All 2D‐STE variables were obtained from 3 consecutive cardiac cycles, and the average values were used for statistical analyses. One cardiac cycle was selected for the baseline correction (from the beginning of the QRS complex to the beginning of the next QRS complex), and the myocardial region of interest for 2D‐STE was determined by manually tracing the endocardial border of the corresponding ventricle. Manual adjustments were made to include the entire myocardium in the region of interest. No filter was applied in this 2D‐STE analysis. As the precise indicators of LV systolic function, LV longitudinal and circumferential strain (LV‐SL and LV‐SC, respectively) were measured using the left apical 4‐chamber view and the right parasternal short‐axis view at the papillary muscle level, respectively. Additionally, as the precise indicator of RV systolic function, RV longitudinal strain (RV‐SL) was evaluated using the RV focus view and the left ventricular 4‐chamber algorithm, as described previously.^9^, ^26^, ^29^, ^30^, ^31^ The RV‐SL was measured as the deformation of only the RV‐free wall (RV‐SL_3seg_) and the entire RV, including the interventricular septum (RV‐SL_6seg_). Each strain value was manually obtained as the absolute negative peak value of each global strain wave automatically generated by the software.

### 2.4 Statistical analysis

All statistical analyses were performed by commercially available software (EZR software version 1.41; Saitama Medical Center, Jichi Medical University).^32^ Categorical data are presented as absolute numbers, and frequencies are presented as percentages. Continuous data are reported as medians with interquartile ranges. For all analyses, *P* < .05 was considered statistically significant.

The normality of the data was evaluated by the Shapiro‐Wilk test. Comparisons among dogs diagnosed with PH, which were classified according to the respiratory diseases (OALD and RLD), dogs with no or low PH probability, and healthy controls were performed by Fisher's exact test for categorical data, 1‐way analysis of variance for normally distributed continuous data, or the Kruskal‐Wallis test for non‐normally distributed data. Multiple comparisons were performed by Tukey's post hoc test for normally distributed data or the Steel‐Dwass test for non‐normally distributed data. Furthermore, the echocardiographic variables used in this study were compared among healthy controls, dogs with no or low PH probability, and dogs diagnosed with PH which were classified according to the presence or absence of PH signs with the same procedure as the 3‐group comparison noted above. Univariable logistic regression analyses were performed to evaluate echocardiographic variables related to the presence of (1) intermediate or high PH probability and (2) PH signs. Odds ratios and 95% confidence intervals (CI) were calculated for each variable. For all analyses, statistical significance was set at *P* < .05.

## 3 RESULTS

### 3.1 Clinical profiles

Fifteen healthy dogs, 10 dogs with no or low PH probability, and 21 dogs diagnosed with PH secondary to respiratory diseases (5 dogs with intermediate PH probability and 16 with high PH probability) were enrolled in this study, consisting of the following breeds: Toy Poodle (n = 7; 15%), Chihuahua (n = 6; 13%), Miniature Dachshund (n = 5; 11%), Shih Tzu (n = 4; 9%), Pomeranian (n = 4; 9%), Mix breed (n = 4; 9%), Pembroke Welsh Corgi (n = 3; 7%), Maltese (n = 2; 4%), Papillon (n = 2; 4%), Pekingese (n = 2; 4%), Yorkshire Terrier (n = 2; 4%), and 1 dog each from 5 other breeds. The clinical characteristics and results of blood gas measurements in the 15 healthy dogs and 31 dogs with respiratory diseases are summarized in Table 1. Age, sex, body weight, systolic, and mean blood pressure did not differ significantly between the respiratory disease groups and the healthy controls (*P* = .052, .264, .625, .284, and .774, respectively). Ten dogs were diagnosed with OALD (tracheal collapse, bronchial collapse, or both of these: n = 7; and brachycephalic obstructive airway disease: n = 3) and 11 with RLD (interstitial lung disease with unstructured interstitial and alveolar pattern, or alveolar pattern: n = 9; pulmonary fibrosis: n = 2); and 2 each with interstitial lung disease and pulmonary fibrosis using computed tomography. Eleven dogs with a high PH probability showed PH signs: syncope (n = 3), syncope + ascites (n = 3), syncope + pleural effusion (n = 2), ascites + pericardial effusion (n = 1), pleural effusion (n = 1), and syncope + ascites + pleural effusion (n = 1). The proportion of dogs with PH signs was significantly higher in dogs with RLD than those with OALD (*P* < .001). Three dogs with RLD showed hypoxemia based on the low value of transcutaneous oxygen saturation (<95%). The partial pressure of carbon dioxide exceeded the upper limit of our laboratory (>45 mmHg) in 5 dogs with respiratory disease (OALD, n = 2; RLD, n = 3). No dogs showed clinical findings of respiratory acidosis based on blood gas measurements. Furthermore, no dogs showed systemic hyper/hypotension, arrhythmia, or systemic disorders that could cause syncope other than respiratory diseases and PH. Although no dogs received cardiac medications, 11 dogs with respiratory disease received some of the following treatments for respiratory diseases: maropitant (n = 8; 26%), antibiotics (n = 6; 19%), bronchodilators (n = 5; 16%), and glucocorticoids (n = 4; 13%). Only 1 dog with PH and RHF had received a pulmonary vasodilator (sildenafil) during the examination. No dogs had sustained arrhythmias that could cause syncope, such as atrioventricular block, sinus arrest, atrial fibrillation, and ventricular tachycardia, based on the repeated 30‐second electrocardiography.

**TABLE 1:** Clinical characteristics, blood pressure, and venous blood gas measurements in this study samples.

Variables	Healthy	No or low PH probability	Intermediate or high PH probability	
			Due to OALD	Due to RLD
n	15	10	10	11
Age (year)	11.4 (9.3‐13.1)	11.8 (9.9‐13.3)	13.3 (12.0‐14.8)	13.2 (11.4‐15.0)
Body weight (kg)	5.7 (4.0‐6.8)	5.7 (2.7‐6.5)	4.5 (2.8‐6.4)	5.2 (4.0‐9.2)
Sex (male/female)	8/7	6/4	3/7	5/6
PH probability (intermediate/high)	—	—	3/7	2/9
PH signs (n, %)*	0 (0%)	0 (0%)	2 (20%)	9 (82%)
Right heart failure (n, %)*	0 (0%)	0 (0%)	1 (10%)	7 (64%)
Syncope (n, %)*	0 (0%)	0 (0%)	2 (20%)	7 (64%)
Systolic blood pressure (mmHg)	123 (117‐131)	138 (121‐144)	137 (116‐151)	130 (110‐142)
Mean blood pressure (mmHg)	104 (89‐112)	110 (101‐122)	109 (106‐124)	105 (93‐118)
Transcutaneous oxygen saturation (%)	100 (99‐100)	98 (94‐103)	100 (98‐100)	97 (93‐99)
pH	—	7.42 (7.40‐7.45)	7.40 (7.37‐7.44)	7.40 (7.37‐7.43)
Partial carbon dioxide pressure (mmHg)	—	41 (33‐45)	41 (40‐46)	43 (38‐47)
Bicarbonate ion (mmol/L)	—	28.9 (23.9‐29.4)	25.1 (24.0‐28.5)	25.0 (22.1‐29.1)
Base excess (mmol/L)	—	3.5 (1.9‐4.6)	3.0 (1.1‐4.8)	0.8 (−3.5 to 4.3)

*Note*: Continuous data are expressed as median (interquartile range). Fisher's exact test was performed for categorical data, and 1‐way analysis of variance or the Kruskal‐Wallis test was performed for continuous variables.

Abbreviations: OALD, obstructive airway/lung disease; PH, pulmonary hypertension; PH signs, clinical signs associated with PH; RLD, restrictive lung disease.

* The variable is significantly different among groups (*P* < .05).

### 3.2 Echocardiographic measurements

Table 2 shows the echocardiographic variables in 15 healthy dogs and 31 dogs classified by respiratory diseases and PH. Trivial mitral valve insufficiency was observed in 3 dogs (2 with OALD and 1 with RLD). There were no significant differences in all echocardiographic variables between healthy controls and dogs with no or low PH probability. Significantly narrowed left heart based on lower LA/Ao and LVIDDN was observed, especially in dogs with PH because of RLD. For indicators of right heart morphology, pulmonary arterial enlargement and RV hypertrophy were observed in dogs diagnosed with PH. Significantly lower RV SV and RV functional indicators, including RV FAC and RV s', were observed in dogs with PH because of RLD. The TR velocity and PVRecho were significantly higher in dogs diagnosed with PH than in those with no or low PH probability. Additionally, PVRecho was significantly higher in dogs with PH because of RLD than in dogs with PH because of OALD (*P* = .048).

**TABLE 2:** Results of echocardiographic variables in dogs classified by respiratory diseases.

Variables	Healthy	No or low PH probability	Intermediate or high PH probability	
			Due to OALD	Due to RLD
n	15	10	10	11
Heart rate (bpm)	131 (102‐146)	113 (87‐134)	112 (94‐133)	112 (85‐156)
LA/Ao	1.3 (1.1‐1.3)	1.3 (1.2‐1.3)	1.2 (1.1‐1.3)	1.0 (0.9‐1.2)* ^,^ ^†^
LVIDDN (cm/kg^0.294^)	1.3 (1.2‐1.4)	1.4 (1.3‐1.6)	1.2 (1.1‐1.3)^†^	1.1 (0.8‐1.3)^†^
LVIDSN (cm/kg^0.315^)	0.7 (0.6‐0.8)	0.8 (0.6‐1.0)	0.6 (0.5‐0.7)	0.6 (0.5‐0.8)
Fractional shortening (%)	44.9 (35.8‐54.0)	40.1 (32.9‐47.6)	44.6 (37.4‐51.4)	36.7 (31.8‐44.4)
LV SV (mL/m^2^)	27.1 (24.4‐31.0)	31.6 (26.4‐34.0)	18.5 (10.4‐22.1)* ^,^ ^†^	18.0 (14.5‐23.4)* ^,^ ^†^
PA/Ao	0.8 (0.8‐0.9)	0.8 (0.8‐0.8)	1.0 (1.0‐1.1)* ^,^ ^†^	1.0 (0.9‐1.0)* ^,^ ^†^
RPAD index	36.8 (29.0‐41.4)	37.1 (33.2‐37.5)	26.1 (23.9‐37.6)	20.1 (13.9‐28.2)* ^,^ ^†^
AT/ET	0.43 (0.39‐0.45)	0.40 (0.35‐0.43)	0.38 (0.29‐0.42)	0.30 (0.25‐0.42)*
RVEDA index (cm^2^/kg^0.624^)	0.8 (0.6‐1.0)	0.8 (0.7‐1.0)	1.0 (0.9‐1.1)	1.1 (0.9‐1.4)*
RVESA index (cm^2^/kg^0.628^)	0.3 (0.3‐0.6)	0.4 (0.3‐0.5)	0.5 (0.5‐0.7)	0.7 (0.5‐1.0)* ^,^ ^†^
RVIDd index (mm/kg^0.327^)	5.7 (5.3‐7.2)	6.0 (5.5‐7.1)	8.0 (6.6‐8.6)*	8.9 (7.2‐10.3)* ^,^ ^†^
RVWTd index (mm/kg^0.254^)	2.5 (2.2‐2.7)	2.8 (2.5‐3.0)	3.2 (3.0‐3.8)*	3.6 (3.1‐4.0)* ^,^ ^†^
RAA index (cm^2^/kg^0.714^)	0.5 (0.4‐0.6)	0.4 (0.4‐0.5)	0.6 (0.5‐0.8)^†^	0.6 (0.4‐0.7)
CVC index (mm/kg^0.245^)	3.7 (3.3‐4.1)	3.7 (3.2‐4.1)	4.2 (3.5‐5.8)	5.0 (3.8‐5.6)^†^
RV FACn (%/kg^−0.097^)	58.6 (52.5‐69.1)	54.9 (50.0‐58.9)	49.3 (41.8‐61.4)	41.4 (39.0‐53.0)*
TAPSEn (mm/kg^0.284^)	5.9 (4.7‐7.7)	5.7 (5.1‐6.3)	4.5 (3.8‐6.7)	4.4 (2.8‐5.0)*
RV s' (cm/s)	11.1 (7.9‐12.5)	10.8 (8.8‐13.8)	7.6 (5.2‐9.8)^*†^	6.5 (4.8‐7.6)* ^,^ ^†^
RV SV (mL/m^2^)	29.7 (25.5‐35.5)	30.3 (25.0‐34.2)	25.3 (21.2‐30.0)	22.0 (15.3‐30.9)*
TR velocity (m/s)	—	2.3 (2.2‐2.6) (n = 6)	4.0 (3.3‐4.3)^†^ (n = 9)	4.5 (3.8‐5.0)^†^ (n = 9)
PVRecho	—	0.5 (0.4‐0.7) (n = 6)	1.6 (1.3‐2.2)^†^ (n = 9)	3.1 (1.9‐3.3)^†,‡^ (n = 9)
LV‐SL (%)	17.2 (14.3‐18.8)	14.9 (13.2‐16.7)	12.8 (11.3‐15.7)*	11.6 (10.5‐12.5)* ^,^ ^†^
LV‐SC (%)	18.3 (16.3‐22.4)	20.9 (19.1‐22.0)	15.5 (14.3‐17.2)^†^	14.7 (14.3‐16.2)* ^,^ ^†^
RV‐SL_3seg_ (%)	26.7 (21.7‐32.2)	28.2 (26.2‐29.5)	21.0 (16.9‐31.6)	16.9 (15.0‐22.0)* ^,^ ^†^ ^,^ ^‡^
RV‐SL_6seg_ (%)	24.9 (19.3‐28.6)	19.0 (18.1‐24.8)	16.7 (13.7‐26.0)	16.1 (11.6‐18.4)* ^,^ ^†^ ^,^ ^‡^

*Note*: Continuous data are expressed as median (interquartile range). Variables with no number of dogs listed were obtained from all dogs in each group.

Abbreviations: AT/ET, acceleration time to ejection time ratio; CVC index, maximal caudal vena cava normalized by body weight; FAC, fractional area change; LA/Ao, left atrial to aortic diameter ratio; LV, left ventricular/left ventricle; LVIDDN, end‐diastolic LV internal diameter normalized by body weight; LVIDSN, end‐systolic LV internal diameter normalized by body weight; LV longitudinal strain; PA/Ao, pulmonary artery to aortic diameter ratio; PH, pulmonary hypertension; PVRecho, pulmonary vascular resistance estimated by echocardiography; RAA index, right atrial area normalized by body weight; RHF, right heart failure; RPAD index, right pulmonary artery distensibility index; RV, right ventricular/right ventricle; RV s', peak systolic myocardial velocity at the lateral tricuspid annulus; RVEDA, end‐diastolic RV area; RVESA, end‐systolic RV area; RVIDd, end‐diastolic RV internal diameter; RVWTd, end‐diastolic RV‐free wall thickness; SV, stroke volume; TAPSE, tricuspid annular plane systolic excursion.

* The value is significantly different from that of healthy controls (*P* < .05).

^†^ The value is significantly different from that of dogs with respiratory disease without PH (*P* < .05).

^‡^ The value is significantly different from that of dogs with PH because of OALD (*P* < .05).

**FIGURE 1:**
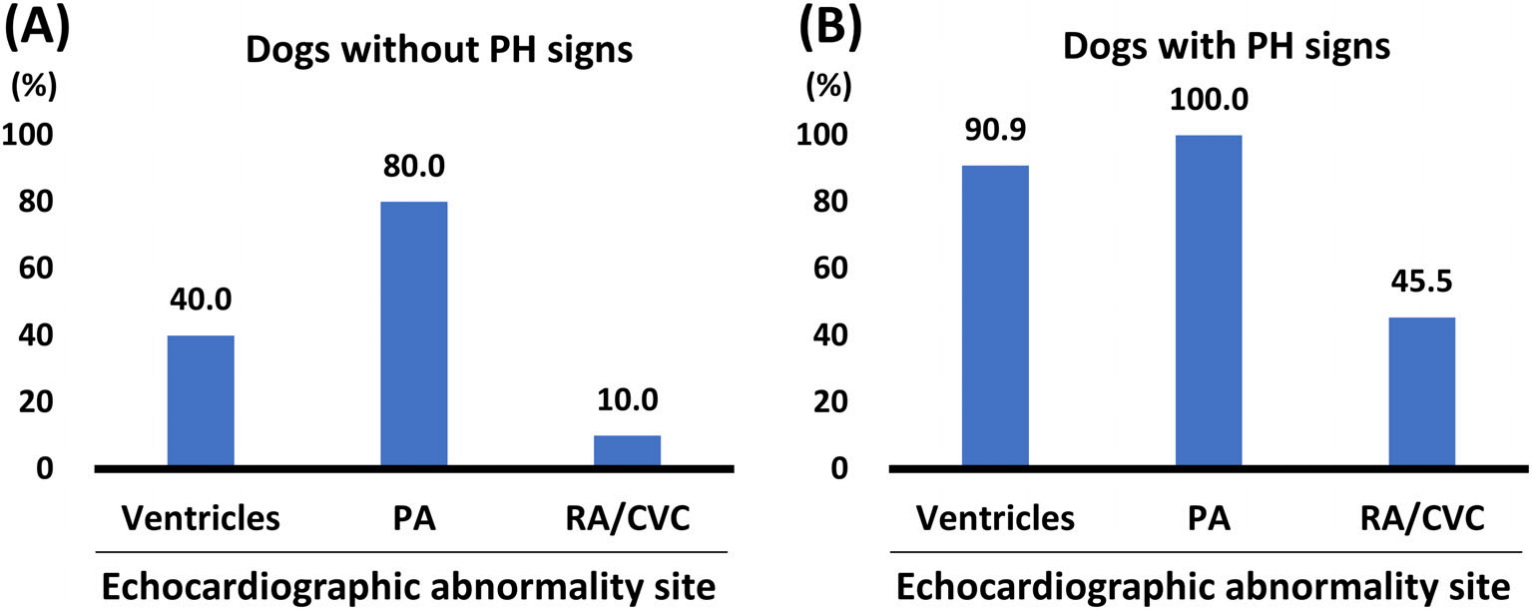
The presence of echocardiographic abnormalities was classified into 3 anatomic sites based on ACVIM consensus in 21 dogs diagnosed with PH: 10 without PH signs (A) and 11 with PH signs (B). ACVIM, American College of Veterinary Internal Medicine; CVC, caudal vena cava; PA, pulmonary artery; PH, pulmonary hypertension; PH signs, clinical signs associated with PH; RA, right atrium.

When dogs diagnosed with PH (ie, intermediate or high PH probability) were classified and compared by the presence of PH signs, significantly higher RV size indicators and lower LV size and RV functional indicators were observed in dogs with PH signs than those of dogs without PH signs (Table 3). Furthermore, dogs with PH signs showed significantly lower LV and RV SV than healthy controls and dogs with no or low PH probability (LV SV: both *P* < .001; RV SV: *P* = .017 and .049, respectively). The TR velocity and PVRecho were significantly higher in dogs diagnosed with PH, especially in dogs with PH signs. Figure 1 shows the proportion of echocardiographic abnormalities in 3 anatomical sites of 21 dogs diagnosed with PH and classified by the presence of PH signs. Echocardiographic abnormalities in ventricles were observed in 14/21 dogs diagnosed with PH (67%; 4/10 dogs without PH signs and 10/11 dogs with PH signs); RV hypertrophy based on increased RVWTd index was observed in all 14 dogs diagnosed with PH; significant RV dilatation, RV dysfunction, and underfilling LV were observed only in dogs with PH signs (n = 10). Echocardiographic abnormalities in the pulmonary artery were observed in 19/21 dogs diagnosed with PH (91%; 8/10 without PH signs and 11/11 with PH signs); increased PA/Ao (>1.0) was found in 18 dogs; decreased RPAD index (<30%) was in 13 dogs (3/10 without PH signs and 10/13 with PH signs); decreased AT/ET (<0.30) was found in 7 dogs (3/7 without PH signs and 4/7 with PH signs). Enlargement of the right atrium and CVC was observed in 6/21 dogs diagnosed with PH (29%; 1/10 without PH signs and 5/11 with PH signs).

**TABLE 3:** Results of echocardiographic variables in dogs classified by the clinical signs of pulmonary hypertension.

Variables	Healthy	No or low PH probability	Intermediate or high PH probability	
			Without PH signs	With PH signs
n	15	10	10	11
Heart rate (bpm)	131 (102‐146)	113 (87‐134)	108 (92‐120)	131 (85‐156)
LA/Ao	1.3 (1.1‐1.3)	1.3 (1.2‐1.3)	1.2 (1.1‐1.3)	1.0 (0.9‐1.2)* ^,^ ^†^
LVIDDN (cm/kg^0.294^)	1.3 (1.2‐1.4)	1.4 (1.3‐1.6)	1.3 (1.2‐1.3)^†^	1.0 (0.8‐1.2)* ^,^ ^†^
LVIDSN (cm/kg^0.315^)	0.7 (0.6‐0.8)	0.8 (0.6‐1.0)	0.7 (0.6‐0.9)	0.5 (0.4‐0.7)^†^
Fractional shortening (%)	44.9 (35.8‐54.0)	40.1 (32.9‐47.6)	42.6 (34.2‐47.9)	38.3 (33.3‐47.0)
LV SV (mL/m^2^)	27.1 (24.4‐31.0)	31.6 (26.4‐34.0)	19.9 (13.9‐25.3)* ^,^ ^†^	17.2 (12.4‐20.8)* ^,^ ^†^
PA/Ao	0.8 (0.8‐0.9)	0.8 (0.8‐0.8)	1.0 (0.9‐1.0)* ^,^ ^†^	1.0 (1.0‐1.0)* ^,^ ^†^
RPAD index	36.8 (29.0‐41.4)	37.1 (33.2‐37.5)	30.2 (25.0‐37.6)	16.8 (13.8‐23.7)* ^,^ ^†^ ^,^ ^‡^
AT/ET	0.43 (0.39‐0.45)	0.40 (0.35‐0.43)	0.38 (0.28‐0.45)	0.32 (0.25‐0.38)*
RVEDA index (cm^2^/kg^0.624^)	0.8 (0.6‐1.0)	0.8 (0.7‐1.0)	0.9 (0.8‐1.0)	1.2 (1.0‐1.4)* ^,^ ^†^
RVESA index (cm^2^/kg^0.628^)	0.3 (0.3‐0.6)	0.4 (0.3‐0.5)	0.5 (0.4‐0.5)	0.7 (0.6‐1.0)* ^,^ ^†^ ^,^ ^‡^
RVIDd index (mm/kg^0.327^)	5.7 (5.3‐7.2)	6.0 (5.5‐7.1)	7.1 (6.3‐8.0)*	9.5 (8.2‐10.3)* ^,^ ^†^ ^,^ ^‡^
RVWTd index (mm/kg^0.254^)	2.5 (2.2‐2.7)	2.8 (2.5‐3.0)	3.1 (2.8‐3.3)* ^,^ ^†^	3.9 (3.4‐4.2)* ^,^ ^†^ ^,^ ^‡^
RAA index (cm^2^/kg^0.714^)	0.5 (0.4‐0.6)	0.4 (0.4‐0.5)	0.5 (0.5‐0.7)	0.7 (0.5‐0.8)^†^
CVC index (mm/kg^0.245^)	3.7 (3.3‐4.1)	3.7 (3.2‐4.1)	4.0 (3.5‐5.1)	5.2 (4.2‐6.5)* ^,^ ^†^
RV FACn (%/kg^−0.097^)	58.6 (52.5‐69.1)	54.9 (50.0‐58.9)	53.1 (47.7‐61.4)	41.3 (39.0‐48.8)* ^,^ ^†^
TAPSEn (mm/kg^0.284^)	5.9 (4.7‐7.7)	5.7 (5.1‐6.3)	4.9 (4.0‐6.7)	4.1 (2.8‐5.0)* ^,^ ^†^
RV s' (cm/s)	11.1 (7.9‐12.5)	10.8 (8.8‐13.8)	7.6 (5.5‐9.8)* ^,^ ^†^	6.5 (4.8‐7.4)* ^,^ ^†^
RV SV (mL/m^2^)	29.7 (25.5‐35.5)	30.3 (25.0‐34.2)	27.6 (23.2‐32.2)	16.8 (15.3‐29.5)* ^,^ ^†^
TR velocity (m/s)	—	2.3 (2.2‐2.6) (n = 6)	3.4 (3.1‐4.0)^†^ (n = 8)	4.6 (4.2‐5.0)* ^,^ ^‡^ (n = 10)
PVRecho	—	0.5 (0.4‐0.7) (n = 6)	1.3 (1.0‐1.7)^†^ (n = 8)	3.1 (2.9‐3.3)* ^,^ ^‡^ (n = 10)
LV‐SL (%)	17.2 (14.3‐18.8)	14.9 (13.2‐16.7)	13.0 (12.0‐15.7)*	11.2 (10.5‐12.2)* ^,^ ^†^
LV‐SC (%)	18.3 (16.3‐22.4)	20.9 (19.1‐22.0)	15.6 (14.7‐17.2)^†^	14.7 (13.7‐16.2)* ^,^ ^†^
RV‐SL_3seg_ (%)	26.7 (21.7‐32.2)	28.2 (26.2‐29.5)	26.0 (20.3‐31.6)	16.9 (14.7‐17.9)* ^,^ ^†^ ^,^ ^‡^
RV‐SL_6seg_ (%)	24.9 (19.3‐28.6)	19.0 (18.1‐24.8)	18.7 (16.0‐26.0)	13.6 (11.6‐16.6)* ^,^ ^†^ ^,^ ^‡^

*Note*: Continuous data are expressed as median (interquartile range). Variables with no number of dogs listed were obtained from all dogs in each group.

Abbreviations: AT/ET, acceleration time to ejection time ratio; CVC index, maximal caudal vena cava normalized by body weight; FAC, fractional area change; LA/Ao, left atrial to aortic diameter ratio; LV, left ventricular/left ventricle; LVIDDN, end‐diastolic LV internal diameter normalized by body weight; LVIDSN, end‐systolic LV internal diameter normalized by body weight; LV longitudinal strain; PA/Ao, pulmonary artery to aortic diameter ratio; PH, pulmonary hypertension; PVRecho, pulmonary vascular resistance estimated by echocardiography; RAA index, right atrial area normalized by body weight; RHF, right heart failure; RPAD index, right pulmonary artery distensibility index; RV, right ventricular/right ventricle; RV s', peak systolic myocardial velocity at the lateral tricuspid annulus; RVEDA, end‐diastolic RV area; RVESA, end‐systolic RV area; RVIDd, end‐diastolic RV internal diameter; RVWTd, end‐diastolic RV‐free wall thickness; SV, stroke volume; TAPSE, tricuspid annular plane systolic excursion.

* The value is significantly different from that of healthy controls (*P* < .05).

^†^ The value is significantly different from that of dogs with respiratory disease without PH (*P* < .05).

^‡^ The value is significantly different from that of dogs with respiratory disease without clinically relevant signs of PH (*P* < .05).

Regarding the 2D‐STE variables, all myocardial segments were successfully used for all 2D‐STE variables (mean ± SD of frame rates: 128 ± 8 frames per second). In this study, all myocardial strain values were significantly lower in dogs diagnosed with PH because of RLD than in healthy controls and dogs with no or low PH probability (*P* = .001 and .004, respectively). Furthermore, dogs diagnosed with PH because of OALD also had lower LV‐SL than healthy controls (*P* = .002) and lower LV‐SC than dogs with no or low PH probability (*P* < .001; Table 2). Meanwhile, when comparing healthy controls and dogs with respiratory disease classified by PH signs, dogs with PH signs had significantly lower values for LV‐SL and LV‐SC than healthy controls and dogs with no or low PH probability. Furthermore, a significantly lower RV‐SL was observed only in dogs with PH signs compared with the other groups (Table 3).

### 3.3 Logistic regression analysis

The results of the univariable logistic regression analysis to detect intermediate or high probability of PH in dogs with respiratory diseases are summarized in Table 4. Decreased LVIDDN, LVIDSN, LV SV, AT/ET, RPAD index, TAPSEn, RV s', LV‐SL, LV‐SC, and RV‐SL_3seg_, and increased PA/Ao, RV size indicators, RAA index, and CVC index were significantly associated with the presence of intermediate probability of PH.

**TABLE 4:** Univariable logistic regression analyses to detect intermediate or high probability of pulmonary hypertension in 31 dogs with respiratory diseases.

Variables	Univariable analysis	
	Odds ratio (95% CI)	*P*
LA/Ao (per 0.1 decrease)	1.60 (0.96‐2.66)	.07
LVIDDN (per 0.1 cm/kg^0.297^ decrease)	1.63 (1.03‐2.59)	.04
LVIDSN (per 0.1 cm/kg^0.315^ decrease)	1.97 (1.14‐3.42)	.02
LV SV (per 1 mL/m^2^ decrease)	1.42 (1.12‐1.81)	.004
PA/Ao (per 0.1 increase)	3.87 (1.45‐13.6)	.006
RPAD index (per 1.0 decrease)	1.58 (1.10‐2.25)	.01
AT/ET (per 0.1 decrease)	3.25 (1.24‐8.56)	.02
RVEDA index (per 0.1 cm^2^/kg^0.624^ increase)	1.75 (1.07‐2.86)	.03
RVESA index (per 0.1 cm^2^/kg^0.628^ increase)	2.45 (1.12‐5.37)	.03
RVIDd index (per 1 mm/kg^0.327^ increase)	3.25 (1.32‐7.96)	.01
RVWTd index (per 0.1 mm/kg^0.254^ increase)	1.29 (1.03‐1.60)	.03
RAA index (per 0.1 cm^2^/kg^0.714^ increase)	1.63 (1.01‐2.62)	.05
CVC index (per 1.0 mm/kg^0.245^ increase)	2.95 (1.07‐8.11)	.04
RV FACn (per 1%/kg^−0.097^ decrease)	1.09 (0.99‐1.21)	.08
TAPSEn (per 1 mm/kg^0.284^ decrease)	2.25 (1.06‐4.80)	.04
RV s’ (per 1 cm/s decrease)	1.96 (1.21‐3.17)	.006
RV SV (per 1 mL/m^2^ decrease)	1.15 (1.00‐1.31)	.05
LV‐SL (per 1% decrease)	1.87 (1.18‐2.97)	.008
LV‐SC (per 1% decrease)	5.77 (1.12‐29.8)	.04
RV‐SL_3seg_ (per 1% decrease)	1.29 (1.06‐1.56)	.01
RV‐SL_6seg_ (per 1% decrease)	1.12 (0.97‐1.31)	.13

Abbreviations: 3seg, only RV‐free wall analysis; 6seg, global RV analysis; AT/ET, acceleration time to ejection time ratio; CI, confidence interval; CVC index, maximal caudal vena cava normalized by body weight; FAC, fractional area change; LV, left ventricular/left ventricle; LVIDDN, end‐diastolic LV internal diameter normalized by body weight; LVIDSN, end‐systolic LV internal diameter normalized by body weight; LV‐SC, LV circumferential strain; LV‐SL, LV longitudinal strain; PA/Ao, pulmonary artery to aortic diameter ratio; PVRecho, pulmonary vascular resistance estimated by echocardiography; RAA index, right atrial area normalized by body weight; RPAD index, right pulmonary artery distensibility index; RV, right ventricular/right ventricle; RV s’, peak systolic myocardial velocity at the lateral tricuspid annulus; RVEDA, end‐diastolic RV area; RVESA, end‐systolic RV area; RVIDd, end‐diastolic RV internal diameter; RV‐SL, RV longitudinal strain; RVWTd, end‐diastolic RV‐free wall thickness; SV, stroke volume; TAPSE, tricuspid annular plane systolic excursion.

Table 5 shows the results of the univariable logistic regression analysis to evaluate the association between echocardiographic variables and the presence of PH signs in healthy dogs and dogs with respiratory diseases. In this study, decreased LA/Ao, LV size indicators, LV SV, RPAD index, RV functional indicators, RV SV, LV‐SL, LV‐SC, and RV‐SL, and increased PA/Ao, RVESA index, RVIDd index, RVWTd index, RAA index, CVC index, TR velocity, and PVRecho were significantly associated with the presence of PH signs.

**TABLE 5:** Univariable logistic regression analyses to detect the presence of clinical signs of pulmonary hypertension in 15 healthy dogs and 31 dogs with respiratory diseases.

Variables	Univariable analysis	
	Odds ratio (95% CI)	*P*
LA/Ao (per 0.1 decrease)	2.05 (1.12‐3.74)	.02
LVIDDN (per 0.1 cm/kg^0.297^ decrease)	1.67 (1.11‐2.51)	.01
LVIDSN (per 0.1 cm/kg^0.297^ decrease)	1.92 (1.09‐3.37)	.02
LV SV (per 1 mL/m^2^ decrease)	1.25 (1.05‐1.47)	.01
PA/Ao (per 0.1 increase)	3.63 (1.18‐11.16)	.02
RPAD index (per 1.0 decrease)	1.31 (1.06‐1.62)	.01
AT/ET (per 0.1 decrease)	2.31 (0.85‐6.33)	.10
RVEDA index (per 0.1 cm^2^/kg^0.624^ increase)	1.36 (0.99‐1.89)	.06
RVESA index (per 0.1 cm^2^/kg^0.628^ increase)	1.74 (1.06‐2.86)	.03
RVIDd index (per 1 mm/kg^0.327^ increase)	2.85 (1.34‐6.05)	.006
RVWTd index (per 0.1 mm/kg^0.254^ increase)	1.72 (1.21‐2.45)	.003
RAA index (per 0.1 cm^2^/kg^0.714^ increase)	2.19 (1.09‐4.39)	.03
CVC index (per 1.0 mm/kg^0.245^ increase)	2.32 (1.20‐4.49)	.01
RV FACn (per 1%/kg^−0.097^ decrease)	1.2 (1.05‐1.38)	.009
TAPSEn (per 1 mm/kg^0.284^ decrease)	3.18 (1.32‐7.64)	.01
RV s' (per 1 cm/s decrease)	1.74 (1.11‐2.75)	.02
RV SV (per 1 mL/m^2^ decrease)	1.21 (1.05‐1.4)	.01
TR velocity (per 1 m/s increase)	1.45 (1.06‐1.98)	.02
PVRecho (per 0.1 increase)	1.32 (1.09‐1.61)	.005
LV‐SL (per 1% decrease)	2.57 (1.21‐5.46)	.01
LV‐SC (per 1% decrease)	1.92 (1.12‐3.3)	.02
RV‐SL_3seg_ (per 1% decrease)	1.39 (1.11‐1.74)	.005
RV‐SL_6seg_ (per 1% decrease)	1.51 (1.08‐2.11)	.02

Abbreviations: 3seg, only RV‐free wall analysis; 6seg, global RV analysis; AT/ET, acceleration time to ejection time ratio; CI, confidence interval; CVC index, maximal caudal vena cava normalized by body weight; FAC, fractional area change; LV, left ventricular/left ventricle; LVIDDN, end‐diastolic LV internal diameter normalized by body weight; LVIDSN, end‐systolic LV internal diameter normalized by body weight; LV‐SC, LV circumferential strain; LV‐SL, LV longitudinal strain; PA/Ao, pulmonary artery to aortic diameter ratio; PVRecho, pulmonary vascular resistance estimated by echocardiography; RAA index, right atrial area normalized by body weight; RPAD index, right pulmonary artery distensibility index; RV, right ventricular/right ventricle; RV s', peak systolic myocardial velocity at the lateral tricuspid annulus; RVEDA, end‐diastolic RV area; RVESA, end‐systolic RV area; RVIDd, end‐diastolic RV internal diameter; RV‐SL, RV longitudinal strain; RVWTd, end‐diastolic RV‐free wall thickness; SV, stroke volume; TAPSE, tricuspid annular plane systolic excursion.

## 4 DISCUSSION

This prospective study investigated RV and LV myocardial function in dogs with PH secondary to respiratory disease. Our study observed deterioration in RV and LV functional indicators based on 2D‐STE in dogs with PH signs. These results suggest that RV and LV function would worsen in dogs with severe PH secondary to respiratory disease and that dogs with respiratory pathology should undergo a comprehensive echocardiographic evaluation. Additionally, our results comparing OALD and RLD suggest that RLD might be associated with more severe PH than OALD.

This study observed significantly higher PA/Ao and RVWTd index values in dogs diagnosed with PH without PH signs compared to healthy controls. Although the ACVIM consensus states that echocardiographic diagnosis of PH should be performed according to TR velocity and abnormalities at various sites of the right heart,^1^ more than half of the dogs diagnosed with PH without PH signs did not show echocardiographic abnormalities other than pulmonary artery enlargement. Some PH dogs without PH signs showed RV hypertrophy based on the RVWTd index; however, RV dilatation was not observed in any cases. This is mainly because of the RV adaptation mechanism against RV pressure overload.^26^, ^33^, ^34^ In previous studies of canine models of chronic embolic PH, the RV could adapt to mild‐to‐moderate RV pressure overload through RV hypertrophy and associated increased RV function, and maintain RV cardiac output. Whereas RV dysfunction attributed to chronic and excessively high RV pressure and volume overload would induce RV dilatation to preserve RV cardiac output (ie, RV maladaptation).^26^ In this study, most PH dogs without PH signs, even those with an intermediate or high probability of PH, had preserved RV function based on RV‐SL and no echocardiographic abnormalities other than the pulmonary artery and RV hypertrophy. Therefore, our results indicate that the abnormality in the pulmonary artery might be the most common echocardiographic finding in dogs with PH secondary to respiratory disease. Additionally, RV dilatation might be a less common finding in subclinical PH secondary to respiratory diseases; in other words, preserved RV function might be associated with a lack of PH signs in dogs with respiratory conditions.

Dogs with PH and PH signs in our study samples showed decreased RV functional indicators and increased RV size indicators compared to healthy controls, dogs with no or low PH probability, and PH dogs without PH signs. Furthermore, a narrowed LV and decreased LV functional indicators, including LV‐SL and LV‐SC, were also observed in dogs with PH signs. These LV morphological indicators possibly reflect a reduced venous return to the left heart attributed to decreased RV SV, which might have worsened LV function and LV SV through the Frank‐Starling mechanism. Additionally, ventricular interdependence might also influence LV function and morphology.^35^, ^36^ Furthermore, worsening LV function and cardiac output because of PH might contribute substantially to cardiac syncope.^37^ Overall, although there has been no study suggesting the importance of evaluating left heart morphology and function in dogs with PH secondary to respiratory diseases, our results indicate that a comprehensive echocardiographic assessment of both the right and left heart should be performed even in dogs with respiratory disease and suspected PH.

Regarding RV functional indicators, only RV s' showed a significant decrease in PH dogs without PH signs, although the other RV functional variables, including 2D‐STE‐derived RV‐SL, showed no significant decrease. In a previous study of healthy dogs, RV s' showed a substantial reduction after atenolol administration compared with the other RV functional variables used in this study.^38^ Therefore, RV s' might be able to detect RV dysfunction associated with PH earlier than other indices. Conversely, RV s' potentially underestimates the RV function of dogs with PH, depending on its angle‐dependency, ventricular interdependence, heart rate, and degree of TR.^31^, ^36^, ^39^ Further studies using right heart catheterization are warranted to confirm the authenticity of the results.

When comparing OALD and RLD, more dogs with RLD had PH signs than those with OALD. Additionally, PVRecho was significantly higher in dogs with RLD than those with OALD. Furthermore, a significantly lower RV‐SL and higher RV area were observed only in dogs with RLD than in healthy controls. In a previous study of dogs with PH secondary to left heart disease, PVRecho was reported as an indicator of PVR and RV performance, which reflects the balance of RV contractility and afterloads.^19^ Although both OALD and RLD could increase pulmonary arterial pressure, our results of PVRecho propose a hypothesis that inflammation and fibrosis around pulmonary vessels associated with RLD might further increase pulmonary vascular resistance, resulting in more severe PH. However, this study had a small sample size and could not perform right heart catheterization in all dogs. Further studies that measure and compare invasive pulmonary vascular resistance in dogs with various respiratory diseases are required to validate the hypothesis.

This study had several limitations. First, we could not perform the right‐heart catheterization for a definitive diagnosis of PH in any dog. Additionally, hypoxemia and the direct causal relationship between respiratory diseases and PH were not clarified because no dogs underwent arterial blood gas measurements. Second, not all dogs underwent computed tomography to diagnose and classify respiratory diseases. Additionally, no dogs have undergone complete differential diagnostic examination to differentiate diseases that might cause syncope, such as magnetic resonance imaging and 24 h Holter monitoring. Third, because 2D‐STE analysis software is proprietary, our results on 2D‐STE variables cannot be compatible with those of the other companies. Finally, the small sample size might have affected the statistical power to detect the difference. Further studies that perform multivariable analysis using a larger population of dogs with respiratory disease and PH are expected to clarify the clinical utility of various echocardiographic variables.

In conclusion, this study revealed that pulmonary arterial enlargement and RV hypertrophy were the common echocardiographic findings in dogs with intermediate or high PH probability secondary to respiratory disease, whereas RV dilatation and dysfunction were observed only in dogs with PH that progressed enough to show clinical signs associated with PH including RHF and syncope. Furthermore, dogs with PH signs showed significantly lower LV and RV functional indicators, including myocardial strain and SV. These findings suggest a comprehensive echocardiographic evaluation is recommended for dogs with respiratory diseases. Dogs with PH because of RLD had significantly higher PVRecho and lower RV‐SL values than those with PH because of OALD, indicating that RLD might induce more severe PH than OALD and requires careful follow‐up.

## CONFLICT OF INTEREST DECLARATION

Authors declare no conflict of interest.

## OFF‐LABEL ANTIMICROBIAL DECLARATION

Authors declare no off‐label use of antimicrobials.

## INSTITUTIONAL ANIMAL CARE AND USE COMMITTEE (IACUC) OR OTHER APPROVAL DECLARATION

All procedures of this study followed the Guidelines for Institutional Laboratory Animal Care and Use of Nippon Veterinary and Life Science University in Japan, and the study was approved by the Ethical Committee for Animal Use of the Nippon Veterinary and Life Science University Veterinary Medical Teaching Hospital, Japan (approval number: R2‐5). Written informed consent authorizing the participation of the dogs in this study was obtained from all dog owners.

## HUMAN ETHICS APPROVAL DECLARATION

Authors declare human ethics approval was not needed for this study.
